# Peripheral Nerve Regeneration Is Independent From Schwann Cell p75^NTR^ Expression

**DOI:** 10.3389/fncel.2019.00235

**Published:** 2019-05-29

**Authors:** Nádia P. Gonçalves, Simin Mohseni, Marwa El Soury, Maj Ulrichsen, Mette Richner, Junhua Xiao, Rhiannon J. Wood, Olav M. Andersen, Elizabeth J. Coulson, Stefania Raimondo, Simon S. Murray, Christian B. Vægter

**Affiliations:** ^1^Danish Research Institute of Translational Neuroscience – DANDRITE, Nordic-EMBL Partnership for Molecular Medicine, Department of Biomedicine, Aarhus University, Aarhus, Denmark; ^2^The International Diabetic Neuropathy Consortium, Aarhus University Hospital, Aarhus, Denmark; ^3^Division of Cell Biology, Department of Clinical and Experimental Medicine, Linköping University, Linköping, Sweden; ^4^Department of Clinical and Biological Sciences, Neuroscience Institute Cavalieri Ottolenghi, University of Turin, Turin, Italy; ^5^Department of Anatomy and Neuroscience, School of Biomedical Science, Faculty of Medicine, Dentistry and Health Sciences, The University of Melbourne, Parkville, VIC, Australia; ^6^School of Biomedical Sciences, Faculty of Medicine, Queensland Brain Institute, The University of Queensland, Brisbane, QLD, Australia

**Keywords:** Schwann cells, p75^NTR^, myelination, regeneration, nerve injury

## Abstract

Schwann cell reprogramming and differentiation are crucial prerequisites for neuronal regeneration and re-myelination to occur following injury to peripheral nerves. The neurotrophin receptor p75^NTR^ has been identified as a positive modulator for Schwann cell myelination during development and implicated in promoting nerve regeneration after injury. However, most studies base this conclusion on results obtained from complete p75^NTR^ knockout mouse models and cannot dissect the specific role of p75^NTR^ expressed by Schwann cells. In this present study, a conditional knockout model selectively deleting p75^NTR^ expression in Schwann cells was generated, where p75^NTR^ expression is replaced with that of an mCherry reporter. Silencing of Schwann cell p75^NTR^ expression was confirmed in the sciatic nerve *in vivo* and *in vitro*, without altering axonal expression of p75^NTR^. No difference in sciatic nerve myelination during development or following sciatic nerve crush injury was observed, as determined by quantification of both myelinated and unmyelinated nerve fiber densities, myelinated axonal diameter and myelin thickness. However, the absence of Schwann cell p75^NTR^ reduced motor nerve conduction velocity after crush injury. Our data indicate that the absence of Schwann cell p75^NTR^ expression *in vivo* is not critical for axonal regrowth or remyelination following sciatic nerve crush injury, but does play a key role in functional recovery. Overall, this represents the first step in redefining the role of p75^NTR^ in the peripheral nervous system, suggesting that the Schwann cell-axon unit functions as a syncytium, with the previous published involvement of p75^NTR^ in remyelination most likely depending on axonal/neuronal p75^NTR^ and/or mutual glial-axonal interactions.

## Introduction

Schwann cells are axon-ensheathing glial cells of the peripheral nervous system (PNS) and are essential in maintaining normal nerve function as well as facilitating nerve repair following injury. Two subtypes of Schwann cells exist in the adult PNS, either myelinating or non-myelinating. The myelinating Schwann cells form a multi-layered myelin sheath around a segment of a single large-caliber axon, spirally wrapping its plasma membrane around the axon. In contrast, non-myelinating Schwann cells surround and segregate groups of several small-diameter nociceptive axons, in a structure called Remak bundles. The development of the PNS into these organized structures has been extensively studied and the identification of essential molecules and signaling pathways established (Jessen and Mirsky, 2005; Richner et al., 2014; Gonçalves et al., 2017). An important group in this context is the family of neurotrophins consisting of nerve growth factor (NGF), brain derived neurotrophic factor (BDNF), neurotrophin-3 (NT-3) and neurotrophin-4/5 (NT-4/5), which binds to two structurally unrelated receptors: the p75 neurotrophin receptor (p75^NTR^) and the tropomyosin receptor kinases (TrkA, -B, and -C). It is generally well accepted that binding of neurotrophins to the Trk receptors mediate survival and differentiation, but the specific functions of p75^NTR^ in the PNS remains elusive. The controversy may partly be attributed to the fact that p75^NTR^ is expressed by both neuronal and glial cell types and furthermore may have very different and even contradictory roles depending on cell type and temporal expression pattern, including survival signaling, cytoskeletal organization as well as the induction of cell death (Chao, 2003; Reichardt, 2006; Meeker and Williams, 2015), providing substantial cellular and molecular diversity of this receptor.

Damage to adult peripheral nerves causes axonal degeneration, myelin degradation and Schwann cell dedifferentiation distal to the injury site in the process of Wallerian degeneration. Interestingly, such injury also induces mechanisms at the cellular level resembling those active during development, including increased neurotrophin synthesis in neurons and Schwann cells which, which is important for guiding and supporting axonal regeneration (Richner et al., 2014). It is well established that p75^NTR^ is expressed by Schwann cells during development and following peripheral nerve injury (Taniuchi et al., 1986; Johnson et al., 1988), where a high expression level is maintained until contact between the (re)growing axons and Schwann cells have been established and (re)myelination initiated (Johnson et al., 1988). In line with this, studies using complete p75^NTR^ knockout (KO) mouse models have found that the lack of p75^NTR^ results in reduced PNS myelination (Cosgaya et al., 2002). However, the mechanism by which p75^NTR^ affects nerve regeneration following injury is unclear. p75^NTR^ has been reported to inhibit motor axonal regeneration (Boyd and Gordon, 2001), be indispensable for motor axonal regrowth (Gschwendtner et al., 2003), to be important for regeneration and remyelination of motor neurons (Tomita et al., 2007), and important for both the number and regrowth of axons in a mixed nerve (Song et al., 2006, 2009). A central tool for the majority of previous studies investigating the role of p75^NTR^ in the PNS has been the complete p75^NTR^ KO mouse models, the exon III deletion (Lee et al., 1992) and the subsequent exon IV model (von Schack et al., 2001). These KO models demonstrate a dramatic PNS phenotype, with an approximately 40–50% reduction in DRG neurons and myelinated axon numbers as well as impaired levels of myelination (Cosgaya et al., 2002). Importantly, these models exhibit loss of p75^NTR^ in Schwann cells, DRG- and motor neurons and the use of such models cannot therefore clarify whether the observed phenotypes result from loss of p75^NTR^ in the Schwann cells, a neuronal subpopulation or both. To complicate matters further, it has subsequently been determined that both models are not complete KOs but retain the expression of an alternative active splice variant (exon III model) or results in a pro-apoptotic fragment (exon IV model) (von Schack et al., 2001; Murray et al., 2003; Paul et al., 2004). In this study, to dissect the role of p75^NTR^ signaling in Schwann cells in the process of nerve regeneration and remyelination following nerve crush injury, we have developed a conditional KO model selectively deleting p75^NTR^ expression in Schwann cells. Our results show that p75^NTR^ deletion in Schwann cells does not affect the structure of the uninjured myelinated or unmyelinated fibers. In response to injury, although axonal regeneration and remyelination appeared unaffected in Schwann cell p75^NTR^ deficient animals, these mice nonetheless exhibited reduced recovery of motor nerve conduction velocity.

## Materials and Methods

### Mouse Model Generation

To generate mice with conditional deletion of p75^NTR^ in Schwann cells, p75^fl/fl^ mice (Boskovic et al., 2014) were crossed to transgenic mice containing the myelin protein zero promoter driving expression of Cre recombinase (Mpz-cre) (sourced from the Jackson Laboratory strain #017927) (Feltri et al., 1999). Upon recombination, the p75^NTR^ genomic DNA flanked by the loxP sites is inverted and, in p75^in/in^ cells, expression of p75^NTR^ is replaced by expression of mCherry (Boskovic et al., 2014). The resulting knockout mice were termed p75^in/in^ Mpz-cre (referred to as SC-p75^NTR^-KO throughout this manuscript). All mice were on a C57BL/6 background and housed in specific pathogen-free conditions at the Melbourne Brain Centre Animal Facility (The Royal Melbourne Hospital, Australia). Animal breeding procedures were approved by The Florey Institute for Neuroscience and Mental Health Animal Ethics Committee and followed the Australian Code of Practice for the Care and Use of Animals for Scientific Purposes.

### Mouse Surgery

For the mouse model of injury, 8–10-week-old female SC-p75^NTR^-KO and p75^NTR fl/fl^ Cre- (control littermates) were used. By this age, the PNS is already mature and injury effects will not be misinterpreted by the developmental process. Anesthesia was induced with isoflurane and a subcutaneous injection of buprenorphine and ampicillin administered prior to surgery, to minimize pain and post-surgical distress. The thigh and legs were shaved, eyes protected from drying and the sciatic nerve exposed at the mid-thigh level by separating the biceps femoris and the gluteus superficialis. After carefully clearing surrounding connective tissue, the left sciatic nerve was crushed with a non−serrated clamp, twice for 15 s. A sham operation was performed similarly at the contralateral side, where the sciatic nerve was exposed and the skin closed immediately after. In both situations, the musculature was prepared with minimum tissue damage to guarantee the ideal conditions for functional recovery.

Mice were cared for in a pathogen-free environment, in a 12 h light/dark cycle and with water and food *ad libitum*. One group of animals was euthanized at post-injured day 15 (*n* = 4 littermates, *n* = 6 SC-p75^NTR^-KO) while the other group was sacrificed 29 days after injury (*n* = 8 littermates, *n* = 6 SC-p75^NTR^-KO). Animals were handled according to the European Union Council Directive and National rules.

### Sensorimotor Analysis

Sensorimotor behavior was analyzed before (0) and 1, 5, 7, 14, and 28 days after injury.

Mechanical allodynia was assessed with the application of a set of calibrated Von Frey filaments (Touch-Test^®^ Sensory Evaluators, North Coast Medical, CA, United States) into the midplantar side of the hind paw until the filament was just bent (bending forces from 0.2 to 2 g). Mice were placed in a Plexiglas cage with mesh flooring and allowed to acclimate for 1 h. The stimulus was repeated five times with each filament and a positive response in three out of five repetitive stimulations stated as the pain threshold. The withdrawal threshold is expressed in grams.

The Hargreaves test was used to measure paw withdrawal latency to a noxious thermal stimulus using a Heat Flow I.R, Radiometer (Hargreaves Apparatus, Cat. #37370, Ugo Basile, Gemonio, Italy). The radiant heat source was kept at 50% (190 mW/cm^2^) in all tested animals that were let to acclimatize for 1 h before the procedure. Hind paws were tested alternately with 5 min between consecutive tests, and five measurements were obtained for each side, that were averaged for a final result. A cut-off of 20 s was established to avoid potential burn injury.

Walking tract analysis was performed to access locomotor functional recovery. Briefly, the mice hind feet were pressed onto a non-toxic ink pad and animals were then allowed to walk through a dark corridor over an A3 white printer paper. The obtained footprints were then measured to calculate the sciatic functional index (SFI) using the empirical equation adapted for mice by Inserra et al. (1998): SFI = 118.9 × [(ETS-CTS)/CTS] − 51.2 × [(EPL-CPL)/CPL] − 7.5, where ETS represents operated experimental toe spread (distance between the first and fifth toes), CTS stands for control toe spread, EPL for operated experimental print length and CPL for control print length (Inserra et al., 1998). Footmarks made at the beginning of the trial were excluded and three analyzable walks were evaluated from each run, for individual step parameter calculation. The pre-injured SFI values (time point = 0) were used as control for comparison. The SFI scores that we processed ranged from 0 to −130, with 0 representing normal or completely recovered nerve function and −100 or more, a non-functional nerve; thus, mice that dragged their toes were arbitrarily assigned a value of −100.

### Nerve Conduction Velocities

Motor (sciatic) and sensory (sural) nerve conduction velocities (NCV) were performed in naïve mice and 29 days injured ones, according to (Oh et al., 2010) using a Viking Quest apparatus (Natus Neurology Incorporated, United States). Briefly, for sural nerve, recording electrodes were placed in the dorsal part of the foot, with supramaximal stimulation at the ankle. Sural sensory NCV (m/s) was calculated by dividing the distance between the recording and stimulating electrodes (mm) by the onset latency (ms) of the sensory nerve action potential after supramaximal antidromic stimulation. Sciatic-tibial motor NCV was recorded by placing electrodes dorsally in the foot and orthodromically stimulating first at the ankle, then at the sciatic notch. The distance between the two sites of stimulation (mm) was then divided by the difference between the two onset latencies (ankle distance and notch distance, ms) to calculate the final sciatic-tibial motor NCV (m/s).

### Immunohistochemistry and Microscopy

Naïve P11 mice were perfused transcardially with 4% paraformaldehyde (PFA), sciatic nerves isolated, frozen and 10 μm cryosections collected. For tissue imaging, frozen sections were incubated with primary antibodies directed against p75^NTR^ (G323A, Promega), βIII-tubulin (G7121, Promega) and contactin-associated protein 1 (Caspr, a kind gift from Professor Elior Peles, Weizmann Institute of Science, Israel), diluted in blocking buffer containing 10% FBS and 0.3% Triton X100 in PBS. Incubation with proper fluorophore-conjugated secondary antibodies (Invitrogen) was followed. PBS was then used to wash the sections that were finally mounted in DAKO mounting medium containing DAPI. Three animals per group were evaluated, and images captured by confocal microscopy (LSM 780, Carl Zeiss, Germany).

### Western Blot Analysis

Sciatic nerves from adult control littermates (*n* = 6) and SC-p75^NTR^-KO (*n* = 6) mice were dissociated in lysis buffer (2 mM CaCl_2_, 1 mM MgCl_2_, 10 mM HEPES, 140 mM NaCl and 1% Triton X-100, pH 7.8, with protease inhibitors from Roche) and centrifuged at 10.000 × *g* for 10 min at 4°C. Total protein concentration was determined using the Bicinchoninic Acid kit from Sigma. Protein lysates were run on 12% SDS-PAGE (20 μg/lane) and electro-blotted for 1.5 h onto polyvinylidenedifluoride (PVDF) filters (Amersham) in 192 mM glycine, 25 mM Tris–HCl, pH 8.0. Membranes were then blocked and incubated overnight at 4°C with the primary antibodies: rabbit anti-p75^NTR^ (1:500, Promega, Cat. #G323A) and mouse anti-β-actin (1:5000, Sigma, Cat. #A5441). Following a washing step, membranes were incubated with horseradish peroxidase (HRP)-conjugated secondary antibodies (1:1000, swine anti-rabbit, Dako, Cat. # P0217; rabbit anti-mouse, Dako # P0260) and blots visualized with the Amersham ECL plus western blotting detection reagents (GE Healthcare) and Fuji film LAS1000. Densitometry was performed with QuantityOne software (Bio-Rad).

### Morphological and Morphometric Analysis

Nerve samples were processed for morphological and morphometrical analysis of myelinated and unmyelinated nerve fibers. Fixation of nerve samples was carried out using 2.5% purified glutaraldehyde and 0.5% sucrose in 0.1M Sorensen phosphate buffer for 2–4 h. Samples were subsequently post-fixed in 2% osmium tetroxide for 2 h at 4°C and dehydrated in a sequence of ethanol from 30 to 100%. After being cleared in propylene oxide, samples were embedded in Glauerts’ embedding mixture of resins containing equal parts of Araldite M and Araldite Harter, HY 964 (Merck, Darmstad, Germany), including 0.5% of the plasticizer dibutyl phthalate and 1–2% of the accelerator 964, DY 064 (Merck). For high-resolution light microscopy, semi-thin transverse sections (2.5 μm) were cut in a Leica Ultracut UCT ultramicrotome (Leica Microsystems, Wetzlar, Germany) and stained with Toluidine blue. Stereology was performed in a DM4000B microscope equipped with a DFC320 digital camera and an IM50 image manager system (Leica Microsystems, Wetzlar, Germany). Systematic random sampling and D-dissector were adopted using a protocol previously described (Geuna et al., 2000, 2004). Total number of myelinated fibers, axon and fiber size, myelin thickness and g-ratio were then determined.

For electron microscopy, ultrathin sections (70 nm thickness) were cut by using an ultramicrotome Leica EM UC7 (Leica, Wien, Austria). Sections were collected onto formvar-coated slot grids and counterstained with uranyl acetate and lead citrate. A 100 kV transmission electron microscope (EM JEM 1230, JEOL Ltd., Tokyo, Japan) was used for qualitative and quantitative examination of the samples. For C-fiber counting, we started at one corner of the fascicle and acquired images of every third microscopic field with 15,000× magnification until 35 images were photographed. C-fibers in the images were counted and their density calculated.

### Schwann Cell Cultures Derived From Sciatic Nerves of Adult Mice

Schwann cell cultures from adult mouse sciatic nerves were prepared as described by Wang and colleagues (Wang et al., 2013), with some alterations to the protocol. In brief, *n* = 16 littermates and *n* = 16 SC-p75^NTR^-KO mice were sacrificed by cervical dislocation prior to sciatic nerve dissection into L-15 medium. The dissected nerves were rinsed in PBS and incubated in Schwann cell culture medium [SCCM; DMEM supplemented with 10% FBS, 2 μM forskolin (Sigma, Cat. #F6886), 10 ng/mL human heregulin-β1 (R&D Systems, Cat. #396-HB) and 50 ng/mL b-FGF (PeproTech, Cat. #100-18B)] for 1 week at 37°C and 5% CO_2_. The media was changed every 2 days. Nerves were then cut into approximately 2 mm and digested for 40 min with a mixture of 0.2% collagenase NB4 Standard Grade (Nordmark Biochemicals, Cat. #S1745402) and 0.2% dispase (Sigma, Cat. #D4693) at 37°C and subsequently triturated and plated onto poly-L-lysine coated 60 mm dishes and incubated for 2 days in SCCM. The mixed cultures where then trypsinized and plated on coverslips at a density of 100,000 cells/coverslip and cultured for additional 2 days before fixation in 4% PFA for further processing.

### Immunocytochemistry and Microscopy of Schwann Cell Cultures

The fixed Schwann cell cultures were permeabilized with PBS containing 0.1% Triton-X 100 for three times 10 min and subsequently incubated in PBS containing 5% donkey serum and 1% BSA to block unspecific binding of the antibodies. The cells were incubated with rabbit anti-p75^NTR^ [1:1500, (Huber and Chao, 1995), Cat. #9651] diluted in PBS containing 1% BSA overnight at 4°C. The samples were incubated 1 h at room temperature the following day before three times 10 min wash in PBS and 4 h incubation with AlexaFlour488 donkey anti-rabbit IgG (H+L) (1:300, Life Technologies, #A21206) diluted in PBS containing 1% BSA. After three times 10 min washing, Hoechst 33258 was used for nuclear staining (1:10.000, Sigma). Sections were then mounted with Dako Fluorescent mounting medium and sealed with nail polish. Images were acquired with a ZEISS Axio Imager 2 microscope (Carl Zeiss, Germany) equipped with a Hamamatsu digital camera (ORCA-flash4.0 digital camera, model C11440-22CU, Hamamatsu Photonics Deutchland GmbH, Germany) and subsequent image analysis performed with ImageJ.

### Statistical Analysis

Statistical comparison of data was accomplished using the Student *t*-test or One/Two-way ANOVA followed by Bonferroni post-test, with Graph Pad Prism software. Quantitative data is reported as mean ± SEM. Statistical significance was established for p^*^ < 0.05, *p*^∗∗^ < 0.01, *p*^∗∗∗^ < 0.001.

## Results

### Targeted Disruption of p75^NTR^ in Schwann Cells

**FIGURE 1:**
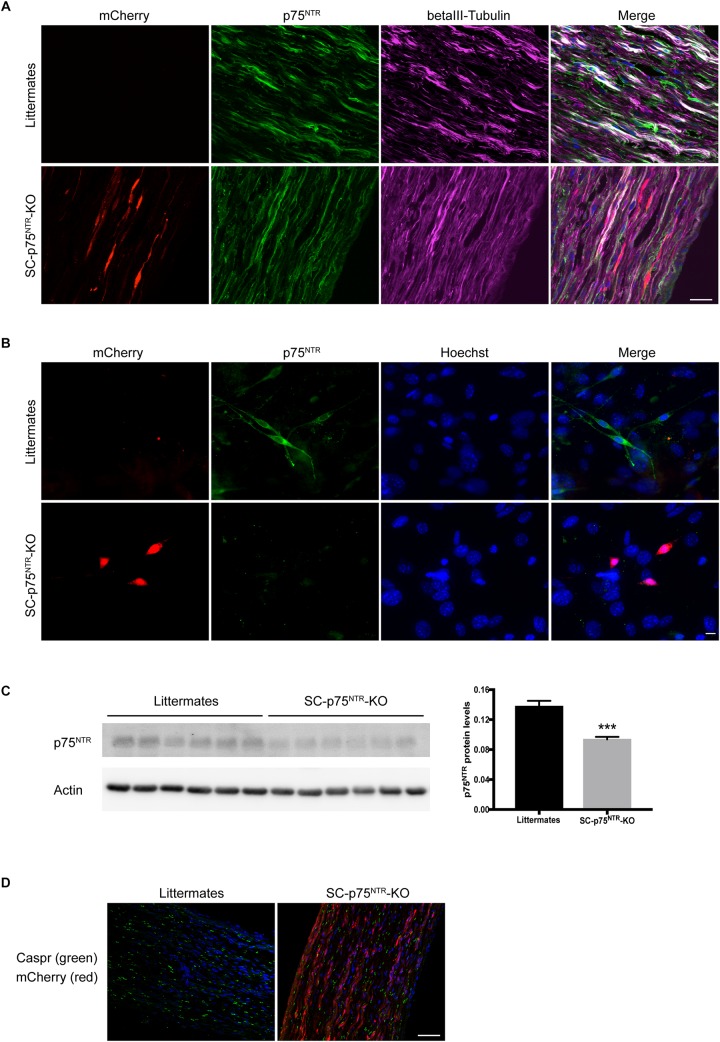
P75^NTR^ inactivation in Schwann cells. **(A)** Double-label immunofluorescence microscopy of p75^NTR^ (green) and βIII-tubulin (pink) in P11 sciatic nerve of SC-p75^NTR^-KO and WT littermates. mCherry emitting fluorescence was identified in channel 568. Nuclei are labeled with Hoechst (blue). The overlap of tubulin and p75^NTR^ reflects axonal p75^NTR^ (white). Note that in the WT, there is a noticeable amount of p75^NTR^ staining that does not overlap with tubulin (green color in the merge), possibly due to p75^NTR^ expression in Schwann cells. Images represent *n* = 4 mice/group; scale bar 25 μm. **(B)** Fluorescence images of cells that were primary cultured from adult mice sciatic nerves, fixed and stained with an antibody against p75^NTR^ (green). mCherry 568 fluorescence was observed in Schwann cells from SC-p75^NTR^-KO but not WT littermates. In the merge panel, it is clear that p75^NTR^ was only present in Schwann cells and not fibroblasts from the WT nerves (cells with bigger nuclei displayed with Hoechst, blue), being completely absent in the SC-p75^NTR^-KO. Scale bar 10 μm; *n* = 16 mice/group. **(C)** P75^NTR^ was determined by immunoblot analysis in extracts of sciatic nerves isolated from adult mice. Quantification of p75^NTR^ ratios by densitometry against levels of β-actin. Data represent the mean ± SEM for six mice/genotype (^∗∗∗^*p* < 0.001 compared with littermate controls). **(D)** Immunofluorescence microscopy of Caspr (green) in naïve P11 sciatic nerves. Note similar intensity and distribution of immunoreactivity throughout the tissue in both genotypes. Nuclei are identified with DAPI (blue) and mCherry visualized in the red channel in SC-p75^NTR^-KO mice. Scale bar, 50 μm; *n* = 4 mice/group.

To generate an *in vivo* model appropriate for investigating the involvement of Schwann cell-expressed p75^NTR^ in peripheral myelination, we developed mice with selective deletion of p75^NTR^ in Schwann cells. For this purpose, mice harboring *loxP* recognition sites in the *Ngfr* gene (Boskovic et al., 2014) were crossed with transgenic mice in which *Cre* recombinase is expressed under the control of the Myelin Protein Zero (P0) promoter (Feltri et al., 1999), to finally produce SC-p75^NTR^-KO mice. The P0 promoter becomes active in Schwann cell precursors at embryonic day 13.5–14.5, which includes cells that develop into both myelinating and non-myelinating Schwann cells but excludes other glial cells and dorsal root ganglion neurons (Feltri et al., 1999, 2002). Therefore, upon introduction of Cre recombinase (as heterozygote Cre^+/–^), the *Ngfr* genomic DNA flanked by loxP sites is inverted and expression of p75^NTR^ in Schwann cells is replaced with that of mCherry, while Cre-negative (Cre^–/–^) littermate controls have unchanged p75^NTR^ expression (Figure 1A). Immunofluorescent staining of sciatic nerves demonstrated a significant amount of p75^NTR^ immunostaining in axons of both SC-p75^NTR^-KO or littermate controls (Figure 1A), confirming that SC-p75^NTR^-KO mice continuously express p75^NTR^ in peripheral neurons/axons. To further substantiate that the inversion strategy resulted in loss of p75^NTR^ protein in Schwann cells, we prepared Schwann cell primary cultures from adult mice. Our results clearly confirmed that SC-p75^NTR^-KO derived Schwann cells presented red mCherry fluorescent signal and no p75^NTR^ immunostaining, with contrary observations in Schwann cells isolated from the littermate controls (Figure 1B). Western blot analysis of lysates isolated from whole sciatic nerves demonstrated an approximately 30% reduction of p75^NTR^ expression in SC-p75^NTR^-KO nerves relative to littermate control nerves (Figure 1C), revealing that the majority of p75^NTR^ protein in adult mouse sciatic nerves is expressed by other cell types, primarily neurons (axons). We did not detect any evidence of altered Mendelian ratios of the SC-p75^NTR^-KO mice relative to littermate controls, and deviation in size or weight was not found between adult mice of different genotypes (data not shown). Finally, there were no indication of any overt neurological abnormalities in the SC-p75^NTR^-KO mice, and Caspr immunostaining in developing nerve (at P11) did not reveal any differences between paranodal junctions in myelinated fibers between genotypes (Figure 1D).

### SC-p75^NTR^-KO Mice Exhibit Mild Behavioral Defects After Nerve Injury

**FIGURE 2:**
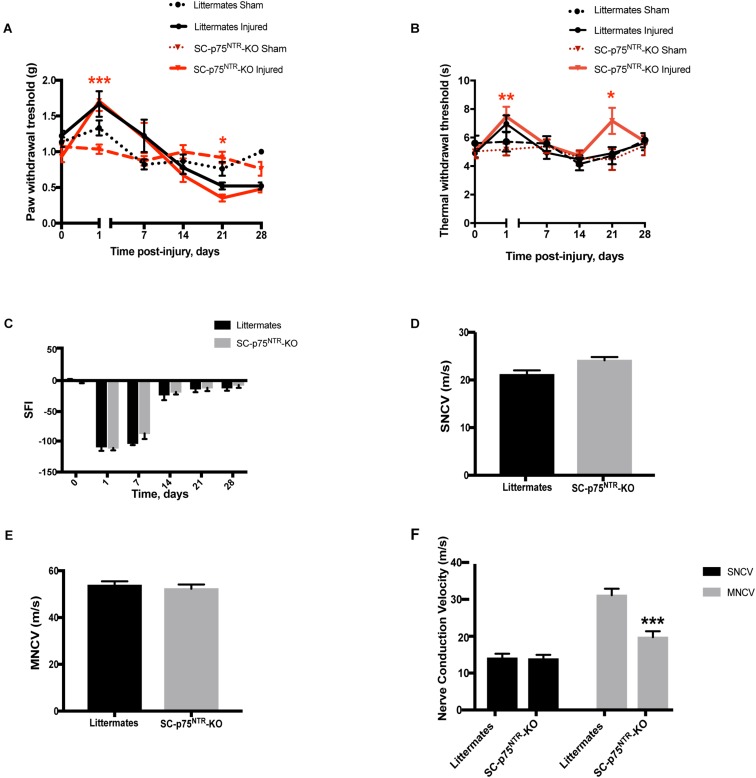
Sensorimotor phenotypic analysis before and after sciatic nerve crush injury. **(A)** Mechanical nociceptive functional recovery evaluated using the Von Frey filaments before and 1, 7, 14, 21, and 28 days after nerve injury. Significant difference noticed between contralateral sham and injured paw at 1- and 21-days post-lesion in the SC-p75^NTR^-KO group, as consequence of nerve injury (^*^p < 0.05, ^∗∗^*p* < 0.01, ^∗∗∗^*p* < 0.001). **(B)** Nociception evaluated with the Hargreaves test at baseline (time = 0), 1, 7, 14, 21, and 28 days after nerve crush injury; (^*^p < 0.05, ^∗∗^*p* < 0.01). **(C)** Locomotor function recovery assessed by walking tract analysis and calculation of SFI at pre-treatment (time = 0), 1, 7, 14, 21, and 28 days after nerve crush injury. Nerve conduction velocity analysis of sural (sensory) **(D)** and sciatic (motor) **(E)** nerves in naïve SC-p75^NTR^-KO and control littermates (*n* = 6 mice per genotype). **(F)** Nerve conduction velocity measurements in both genotypes 29 days after injury (^∗∗∗^*p* < 0.001; *n* = 6 SC-p75^NTR^-KO and *n* = 8 littermates).

To investigate the sensorimotor phenotype of SC-p75^NTR^-KO mice, we initially evaluated sensory profiles by von Frey filaments (mechanical sensitivity) and Hargreaves test (thermal/heat sensitivity), as well as motor recovery by walking tract analysis. Tests were performed as pre-injury baseline (time point = 0) and at 1, 7, 14, 21, and 28 days following sciatic crush injury. Baseline levels were equivalent between sham-operated SC-p75^NTR^-KO and littermate controls for both Von Frey and Hargreaves tests (Figures 2A,B). As expected, both genotypes demonstrated reduced sensitivity from day 1 post-injury, confirming destruction of sensory axons by the crush procedure. Mechanical sensitivity increased over the following period for both genotypes, likely reflecting axonal regeneration and accompanying reinnervation of target tissues. The recovery profile was largely identical for the two genotypes, reaching baseline levels at day 14 and with a non-significant tendency of increased sensitivity in the injured paw. For the SC-p75^NTR^-KO we observed a small but significant transient decrease in mechanical threshold at day 21 (relative to the contralateral uninjured paw), however, this effect was absent at day 28 (Figure 2A). Hargreaves test demonstrated a pattern largely identical to that of the von Frey test; 1-day after injury, both SC-p75^NTR^-KO mice and littermate controls experienced reduced sensitivity to heat-induced noxious stimulus that recovered to baseline levels by day 7. We again observed a transient reduction in sensitivity 21 days following nerve crush in the SC-p75^NTR^-KO mice, which was absent by day 28 (Figure 2B). Recovery of motor function was determined by assessing the SFI at the same time points as for the sensory tests. No differences were found at day 0 or any other time point after sciatic nerve crush injury among the two mice groups (Figure 2C).

Electrophysiological properties of the sciatic nerves were evaluated by measurements of sensory and motor nerve conduction velocity (SNCV and MNCV, respectively) at 29 days post nerve damage, as a terminal endpoint. Nerves from naïve SC-p75^NTR^-KO mice and littermate controls displayed identical SNCV (Figure 2D) and MNCV (Figure 2E). As expected, SNCV and MNCV were significantly reduced following the injury. However, although we did not detect any difference in post-injury SNCV between SC-p75^NTR^-KO mice and littermate controls, SC-p75^NTR^-KO mice exhibited a further 36% reduction of post-injury MNCV relative to littermate controls (Figure 2F), pointing toward ion leakage or/and structural defects in the largest myelinated axons.

### Conditional Deletion of p75^NTR^ in Schwann Cells Has No Impact on Peripheral Myelination or Axon Regeneration Following Nerve Injury

**FIGURE 3:**
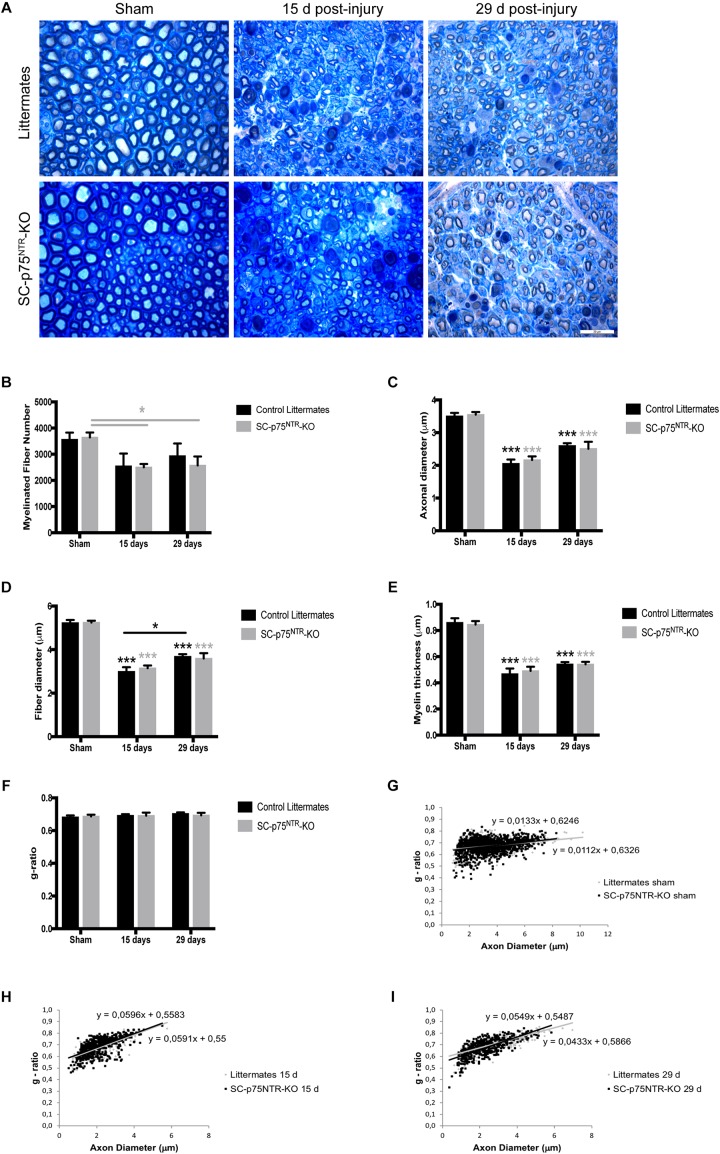
Ablation of p75^NTR^ in Schwann cells has no impact on re-myelination after nerve injury. **(A)** Representative light microscopy images of toluidine-blue stained semithin transverse sections of sham contralateral and distal stumps from crush injured nerves, 15- and 29-days post-lesion. Scale bar 20 μm. **(B–G)** Histograms obtained from the morphometrical analysis of the myelinated fibers. **(B)** Total number of myelinated fibers, **(C)** axon diameter, **(D)** fiber diameter, **(E)** myelin thickness, and **(F)** g-ratio. Scatter plots showing g-ratio of individual myelinated axons as a function of axon diameter in contralateral shams **(G)** or injured nerves at time points of 15 **(H)** and 29 days post lesion **(I)**. Numbers for quantifications were as follows: *n* = 11 sham, *n* = 6 at 15 days, and *n* = 6 at 29 days for SC-p75^NTR^-KO; *n* = 9 sham, *n* = 3 at 15 days, and *n* = 6 at 29 days for littermates.

The observation of decreased MNCV in the injured SC-p75^NTR^-KO mouse model could be due to variation in Schwann cell remyelination of the damaged axons since appropriate re-myelination after peripheral nerve injury has previously been attributed to p75^NTR^ (Song et al., 2006; Tomita et al., 2007). We therefore evaluated nerve fiber morphology in semi-thin cross-sections of sciatic nerves using light microscopy. Representative images of sciatic nerves from SC-p75^NTR^-KO and littermate controls, 15 and 29 days after injury, together with respective non-lesioned nerves (contralateral sham) are illustrated in Figure 3A.

Naïve sciatic nerves from SC-p75^NTR^-KO mice appeared normal, with axons of various diameters present in proportions that appeared similar to those observed in littermate controls and with similar total numbers of myelinated axons (Figures 3A,B). Features of myelinated axonal degeneration consistent with axonal loss were observed in both SC-p75^NTR^-KO and littermate controls after injury, with a similar decrease in axon number (Figure 3B) in mice of both genotypes, however, only statistically different in the SC-p75^NTR^-KO mice. Mean of axonal diameter (Figure 3C), fiber diameter (Figure 3D) or myelin thickness (Figure 3E) were unchanged between genotypes both before and following crush injury, but with an injury effect over time, compared with the respective shams, as expected. Quantification of acquired light microscopy images (with about 100 fibers analyzed per animal) revealed no differences in averaged g-ratios (Figure 3F) or in g-ratios of individual fibers vs. axon diameters, as illustrated by scatter plots of sham and injured nerves, 15 or 29 days after damage (Figures 3G–I).

**FIGURE 4:**
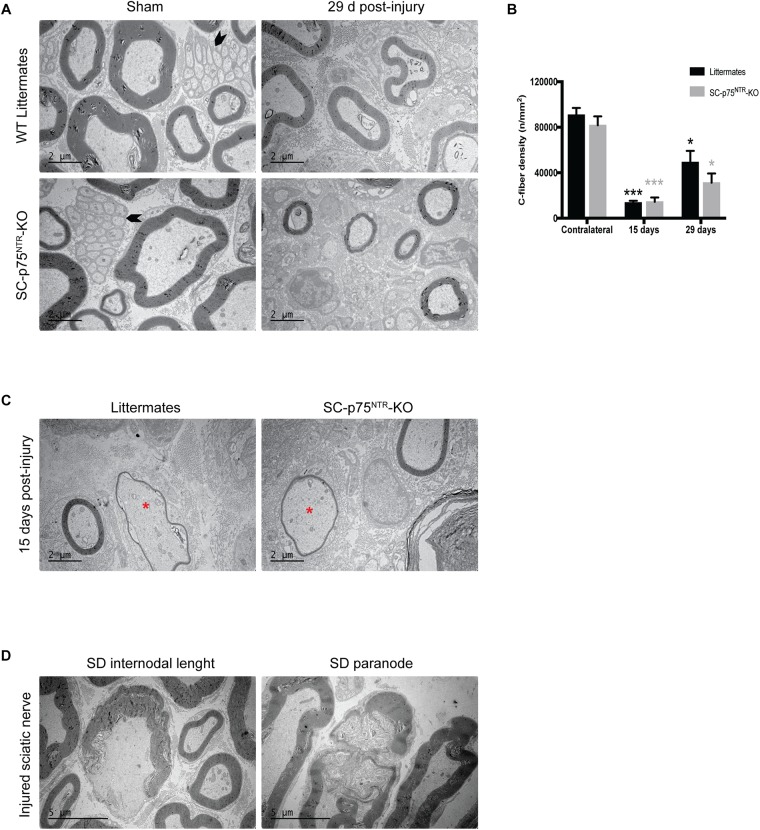
Trend toward decreased C-fiber density in injured nerves from mice lacking Schwann cell p75^NTR^ expression. **(A)** Representative images of ultrathin transverse sciatic nerve sections from sham contralateral nerves (left panel) and 29 days post injury distal stumps (right panel) of the two experimental groups. Arrowheads identify C-fibers. Scale bar 2 μm. **(B)** Density of C-fibers expressed as number of unmyelinated axons per square millimeter (n/mm^2^) Numbers for quantifications were as follows: *n* = 12 sham, *n* = 6 at 15 days, and *n* = 4 at 29 days for SC-p75^NTR^-KO; *n* = 9 sham, *n* = 3 at 15 days, and *n* = 4 at 29 days for littermates. **(C)** Electron micrographs showing re-myelinated fibers (asterisk) in both SC-p75^NTR^-KO and control WT littermates, 15 days after sciatic nerve crush injury. **(D)** Representative pictures of segmental demyelination observed in both strains after sciatic nerve injury, both at internodal and paranodal regions. Scale bar 5 μm.

Proceeding with ultrastructural EM analysis of sciatic nerve transverse sections, we found that the uninjured contralateral nerve of both SC-p75^NTR^-KO and control mice was dominated by large and medium-sized myelinated axons. Unmyelinated axons (C-fibers) occurred in large groups embedded in Schwann cells, forming Remak bundles (Figure 4A, left panel). The endoneurium contained several blood vessels, fibroblasts, and a few mast cells and macrophages. No variance in C-fiber density was observed between the two mice cohorts (Figure 4B). Pathology was evident in the injured ipsilateral side of mice of both genotypes 15 days after crush, with most large myelinated axons being absent and several medium-sized remyelinated axons present in the endoneurium (Figure 4C). At days 15 and 29 post injury we observed myelin debris, multi-vesicular bodies (MVB) and osmophilic and osmophobic fat droplets in the perineurium, endoneurium, Schwann cells, macrophages, fibroblasts and pericytes in all injured nerves. Segmental demyelination of the internodal length and paranode (Figure 4D) was a prominent feature of injured nerves from both WT and SC-p75^NTR^-KO mice. Unmyelinated axons were very sparse and predominantly appeared solitary rather than in high numbers in Remak bundles in the Schwann cell cytoplasm (Figure 4A, right panel). No obvious morphological differences were found between control littermates and SC-p75^NTR^-KO mice; however, although not statistically significant, the mean C-fiber density in SC-p75^NTR^-KO mice tended to be slightly decreased compared to that of littermate controls, 29 days after injury (Figure 4B). This result suggests that lack of p75^NTR^ expression in Schwann cells can possibly alter their molecular signaling profile, affecting glial-axon communication and slowing down the C-fiber regenerative process.

## Discussion

In this study, to elucidate the *in vivo* function of p75^NTR^ in Schwann cells during re-myelination and regeneration after peripheral nerve injury, we generated a new mouse model for the conditional deletion of p75^NTR^ in Schwann cells. Through functional testing, morphological and morphometrical analyses of SC-p75^NTR^-KO mice, we found that ablation of p75^NTR^ in Schwann cells correlated with a reduced motor nerve conduction velocity but had no impact regarding remyelination or axonal growth after sciatic nerve crush injury.

p75^NTR^ is widely expressed in the nervous system during development and has even been considered as a neural crest marker (Wislet et al., 2018). Although p75^NTR^, also called NGF receptor, has no intrinsic catalytic activity, it interacts and modulates the function of TrkA, TrkB, and TrkC, providing substantial cellular and molecular diversity for regulation of neuron survival, neurogenesis, immune responses and processes that support neural function (McGregor and English, 2019). In Schwann cells, p75^NTR^ is expressed by the immature type during development and remains being expressed by the mature non-myelinating Schwann cells in the adult. In contrast, myelinating Schwann cells are not immunoreactive for p75^NTR^ (Jessen and Mirsky, 2005). Because p75^NTR^ is highly down-regulated as the nervous system matures, a powerful tool to induce expression of this receptor is the dedifferentiation of Schwann cells by induced nerve injury. Several reports demonstrated dramatic upregulation of p75^NTR^ in response to injury or disease, in both the PNS and CNS (Meeker and Williams, 2015). However, these previous studies focusing on the roles of p75^NTR^ in development and nerve regeneration have utilized the complete p75^NTR^ KO mouse model, resulting in loss of p75^NTR^ in both Schwann cells as well as in a DRG neuron subpopulation (Lee et al., 1992; von Schack et al., 2001).

Two complete p75^NTR^ KO models have been developed, both of which demonstrate a dramatic PNS phenotype with approximately 50% reduction in neuron/myelinated axon number as well as reduced myelination of the remaining axons. The first model was constructed by deleting exon III of the *Ngfr* gene (Lee et al., 1992), however, it later turned out that this model produces an alternatively spliced isoform of p75^NTR^ (s-p75^NTR^), producing a protein that lacks the portion of the extracellular domain responsible for neurotrophin binding but with its intracellular death-signaling moiety intact (Murray et al., 2003). Complicating the picture, it appears that the genetic background of the mouse also impacts on the phenotype as expression of the s-p75^NTR^ splice variant demonstrated a strain dependent expression level (Naumann et al., 2002). This finding triggered the development of another KO model, the exon IV KO model (p75^NTR^ExonIV) which does not express the s-p75^NTR^ variant. The p75^NTR^ExonIV mice display a more severe phenotype than the previous p75^NTR^exonIII mutants, including a larger reduction in the number of DRG neurons and Schwann cells, vascular system defects, and approximately 40% of p75^NTR^ExonIV mice do not survive beyond the perinatal period (von Schack et al., 2001). It was, however, soon determined that the p75^NTR^ExonIV model express a p75^NTR^ gene product encoding a truncated protein with an apparent molecular weight of 26 kDa (Paul et al., 2004). The utilization of mouse models with a general targeting profile (including both neurons and Schwann cells) combined with the incomplete nature of the p75^NTR^ deletions and the difficulties in delineating the impact of the genetic background, all complicates a rigorous interpretation of previous findings on the role of p75^NTR^ in specific PNS cell subpopulations.

In the present study, the conditional deletion of p75^NTR^ in Schwann cells is driven by the P0 promotor, expressed in Schwann cell precursors early in development (around E14), thus silencing p75^NTR^ expression in both myelinating and non-myelinating Schwann cells types (Feltri et al., 1999). Several studies support a key role for p75^NTR^ in the myelination process (Chan et al., 2001; Cosgaya et al., 2002; Tolwani et al., 2004). Surprisingly, our present results demonstrate that depleting the myelinating Schwann cell subpopulation of p75^NTR^ during the myelination process does not compromise myelin sheet formation nor motor function in the adult naïve SC-p75^NTR^-KO mice.

Boskovic et al. (2014) have not detected any splice variant at the transcriptional or protein levels when developing the p75^in/in^ mouse line used in our study. The fact that we see no neuronal phenotype (reduction in axon number) supports that the model is “clean” from the splice variants found in the p75^NTR^ExonIII and p75^NTR^ExonIV models (which display dramatic PNS phenotypes). Besides neurons and Schwann cells, other cells types have been reported to express p75^NTR^ such as macrophages (Wong et al., 2010), endothelial cells (Tanaka et al., 2004), white fatty tissue (Peeraully et al., 2004), and fibroblasts (Palazzo et al., 2011). Whether such cell types in the sciatic nerve also express p75^NTR^ remains to be determined but this (along with continued axonal expression) may explain the remaining expression of p75^NTR^ in the sciatic nerve upon deletion of Schwann cell p75^NTR^. Our *in vitro* cultures of mouse Schwann cells reveal that these cells are indeed devoid of p75^NTR^ (and positive for mCherry, confirming Cre-activity in these cells).

Due to the pleiotropic roles of p75^NTR^, it is not surprising that its role in regeneration following nerve injury has been controversial. While Song et al. observed reduced regeneration in the p75^NTR^ KO model, work from Scott and Ramer reported that p75^NTR^ expressed by Schwann cells is actually deleterious for nerve regeneration. The mechanism suggested was that increased Schwann cell p75^NTR^ expression might sequester endogenous neurotrophins and thus reduce their availability for axonal Trk signaling (Scott and Ramer, 2009); a notion supported by the enhanced regeneration of injured peripheral motor axons in mice lacking the neurotrophin-binding domain of p75^NTR^ (Boyd and Gordon, 2001). In contrast with this observation, another study using transplantation of p75^NTR^-deficient Schwann cells to injured nerves from nude mice found that a lack of glial p75^NTR^ had a negative impact in the regeneration of motor neurons (Tomita et al., 2007). This model is closer to ours in terms of Schwann cell selectivity, however, the fact that they used nude mice may complicate comparisons since other mechanisms might have been activated by the inhibited immune system, which consequently altered the Schwann cell-axon signaling profile. Nevertheless, like in our study, no difference in the total number of myelinated fibers and fiber density was observed, but a significant decrease in motor nerve conduction velocity was detected (Tomita et al., 2007). Myelin affects nerve conduction velocity and in contrast with our observations, p75^NTR^ null Schwann cell-grafted mice displayed a significant decrease in myelin thickness (Tomita et al., 2007), which could explain the altered motor nerve conduction profile. Yet, this is not always the case; for example, in mouse models of diabetic neuropathy, decreased nerve conduction velocity is often not accompanied by demyelination or fiber loss (Hinder et al., 2017). The reason why this happens is not really understood, but several factors are known to alter nerve conduction velocity in addition to motor axon loss or decreased myelin thickness, such as length of nodal gap (Arancibia-Cárcamo et al., 2017), nodal axonal hydropic swelling (Kolaric et al., 2013), distribution of sodium channels, defects in the Na^+^/K^+^ ATPase (Freeman et al., 2016), impaired Schwann cell exocytosis (Chen et al., 2012), mitochondrial fiber deficiency (Viader et al., 2011; Bala et al., 2018) or reduced endoneurial nutritive blood flow (Coppey et al., 2001). We speculate that in our model, the segmental demyelination of the internodal length and paranode in the SC-p75^NTR^-KO injured nerves leads to leakage of ions from the axons culminating with slower motor nerve conduction velocities, throughout a mechanism that still needs further clarification but that might involve alterations in axonal sodium channels, molecular signaling or composition, and differential regulation of myelin production and repair (Taveggia et al., 2010). Thus, it is fair to speculate that p75^NTR^ might have a role in internode length and/or nodal composition after injury. In line with this, the regulation of neuronal form and function by Schwann cells has been found to be mediated by different forms of intercellular communication (Orellana et al., 2012; Samara et al., 2013), and recent findings suggest the occurrence of lateral molecular cargo transfer to axons mediated by exosomes secreted from Schwann cells (Lopez-Verrilli and Court, 2012). This mechanism has been poorly explored to date, but recent papers have described Schwann cell secreted exosomes being incorporated into axons and increasing neurite sprouting (Court et al., 2008; Lopez-Verrilli et al., 2013). These findings open a new dimension to the degree of intercellular interactions and the idea of a functional nerve syncytium. Deletion of p75^NTR^ in Schwann cells had no apparent effect on basic sciatic nerve structure (developmental effects) including the number or density of myelinated and unmyelinated axons, myelination (g-ratio), nor on regeneration upon crush injury. This is somewhat surprising, considering the observations that p75^NTR^ is highly expressed in Schwann cells during development and the regenerative process. It is now accepted that neurons can synthesize proteins locally in axons and dendrites, and that this localized translation is required for neuronal homeostasis (Jung et al., 2012). Perhaps neurons/axons develop compensatory mechanisms to balance and counteract the absence of p75^NTR^ Schwann cell expression, altering e.g., vesicle cargo and the glial-axon communication process?

A mouse line carrying a conditional knockout allele for p75^NTR^ (p75^NTR^ExonIV–VI) was previously generated to investigate its functions in DRG neurons *in vivo* (Bogenmann et al., 2011). The allele was designed such that a complete and conditional knockout could be achieved without the molecular complexities observed in mice with either the p75^NTR^ExonIII (von Schack et al., 2001) or the p75^NTR^ExonIV allele (Paul et al., 2004). While otherwise normal in size (contrasting the ExonIV model), p75^NTR^ExonIV–VI mice displayed an abnormal hind limb “clenching” phenotype similar to that seen in p75^NTR^ExonIII and p75^NTR^ExonIV mice, suggesting that this common neuropathic phenotype was at least in part due to the impaired function of p75^NTR^ specifically in neural crest cells. In addition, there were far fewer small (unmyelinated and lightly myelinated) diameter axon bundles in the mutant nerves (Bogenmann et al., 2011). Another study using stereological counting demonstrated that p75^NTR^ExonIII mice display a 52% loss of DRG neurons, with the loss of small nociceptors being larger than the loss of large proprioceptors (57 vs. 39%, respectively), and these data were supported by similar results obtained by nerve fiber counts of unmyelinated vs. myelinated axons (Gjerstad et al., 2002). This could either reflect that p75^NTR^ expression occurs predominantly in small nociceptors or that it is most important for non-myelinating Schwann cells to support axon survival, homeostasis and growth. The fact that non-myelinating Schwann cells express p75^NTR^ in adult mice in contrast to the myelinating type also fits well within this observation. Recently, one study indicated that neuron-specific deletion of p75^NTR^ at E12.5 was insufficient to impact on neuronal survival during embryogenesis whereas 20% of DRG neurons were lost in adult mice, suggesting that neuronal p75^NTR^ functions only postnatally in sensory neuron diversification (Chen et al., 2017). In our Schwann cell conditional p75^NTR^ KO mouse model, we found a trend for reduced preservation of unmyelinated fibers in the sciatic nerve 29 days after injury. The crush injury model is a model of axonotmesis, in which axons are disrupted but the connective tissue and the Schwann cell basal lamina remains intact. When this crush model is applied in rodents, axonal regeneration is remarkably efficient and function is restored in 3–4 weeks (Jessen et al., 2015). Thus, it would be predictable that potential early or late differences regarding remyelination and axon regeneration would be detected by evaluation of nerve morphology and morphometry at both 15- and 29-days post-crush injury. Nevertheless, whether different outcomes could have been found at even earlier time points after nerve injury remains to be investigated.

In summary, the influence that p75^NTR^ exerts upon the regenerative and remyelinative processes in the PNS remain controversial, with conflicting findings in DRG- and motor neurons (Tomita et al., 2007; Scott and Ramer, 2009) and a lack of clarity around Schwann cell and neuronal influences. Through utilization of a Schwann cell specific p75^NTR^ knockout strategy, we demonstrate that Schwann cell expression of p75^NTR^ is not important for either sciatic nerve regeneration and remyelination following crush injury, but does exert important influences upon recovery of motor nerve function.

## Ethics Statement

This study was carried out in accordance with the recommendations of The Florey Institute for Neuroscience and Mental Health Animal Ethics Committee and Danish regulations. The protocol was approved by the Australian Code of Practice for the Care and Use of Animals for Scientific Purposes and the Danish Animal Experiments Inspectorate under the Ministry of Environment and Food.

## Author Contributions

NG, SSM, and CV designed the experiments. NG, SM, MS, MU, MR, and RW performed the experiments. NG, SM, JX, OA, EC, SR, SSM, and CV interpreted the results, contributed with reagents, materials, and analysis tools. NG, MU, and CV wrote the manuscript. All authors read and helped to complete the manuscript.

## Conflict of Interest Statement

The authors declare that the research was conducted in the absence of any commercial or financial relationships that could be construed as a potential conflict of interest.
